# Association between sleep duration and hypertension risk in patients with obstructive sleep apnea

**DOI:** 10.1038/s41533-025-00429-7

**Published:** 2025-04-28

**Authors:** Yi Wang, Xi Xi Chen, Fang Ying Lu, Ya Ru Yan, Shi Qi Li, Liu Zhang, Ying Ni Lin, Qing Yun Li

**Affiliations:** 1 Department of Respiratory and Critical Care Medicine, Ruijin Hospital, Shanghai Jiao Tong University School of Medicine, Shanghai, 200025 China; 2 Institute of Respiratory Diseases, Shanghai Jiao Tong University School of Medicine, Shanghai, 200025 China; 3 College of Health Science and Technology, Shanghai Jiao Tong University School of Medicine, Shanghai, 200025 China

**Keywords:** Health care, Disease prevention, Preventive medicine

## Abstract

Obstructive sleep apnea (OSA) is a well-established risk factor for hypertension, with sleep duration being a modifiable factor influencing this risk. However, sleep misperception among OSA patients makes it unclear how subjective and objective sleep duration are associated with the prevalence and incidence of hypertension in this population. This study aims to examine these associations using data from the Sleep Heart Health Study cohort. Participants with OSA (apnea-hypopnea index ≥ 15 events/hour) were categorized based on objective sleep duration from polysomnography (PSGTST) and subjective sleep duration (morning-reported sleep time, AMTST; habitual sleep time, HABTST). Hypertension prevalence was assessed at baseline, while hypertension incidence was evaluated during a five-year follow-up. Multivariable logistic regression and Poisson log-link models were employed to explore the association between sleep duration and hypertension risk, with restricted cubic splines used to assess nonlinear trends. Among 2574 participants with OSA, 1263 had hypertension at baseline. Over 5.25 years, 376 of 1001 patients without baseline hypertension developed hypertension. Shorter PSGTST was linearly associated with higher hypertension prevalence (*p* = 0.009) and incidence (*p* = 0.024). HABTST showed a U-shaped relationship with hypertension prevalence, while AMTST was not significantly associated with either outcome. In patients with OSA, objective sleep duration is linearly and inversely associated with both the prevalence and incidence of hypertension, showing stronger and more consistent associations than subjective sleep duration measures. These findings highlight the value of incorporating objective sleep assessment in evaluating hypertension risk in this population.

## Introduction

Obstructive sleep apnea (OSA) is a common sleep disorder characterized by recurrent upper airway obstruction during sleep, leading to intermittent hypoxia, sleep fragmentation, and increased cardiovascular strain^1,2^. Substantial evidence has firmly established OSA as an independent risk factor for hypertension—a major modifiable driver of cardiovascular disease that imposes significant global morbidity, mortality, and healthcare burdens^3–7^. Understanding the factors that contribute to hypertension in OSA patients is critical for improving cardiovascular outcomes in this high-risk population.

Sleep duration has emerged as a key determinant of blood pressure regulation. Both short sleep and long sleep have been associated with an elevated risk of hypertension, and recent Mendelian randomization studies confirm the causal role of short sleep duration in hypertension^8,9^. However, most previous studies have relied on self-reported sleep duration, which is subject to recall bias and inaccuracies. This issue is particularly pronounced in OSA patients, who often exhibit discrepancies between subjective and objectively measured sleep duration. Notably, individuals with coexisting OSA and insomnia tend to underestimate their sleep duration compared to polysomnography (PSG) measurements^10–13^, whereas those with more severe OSA are more likely to overestimate it^13^. Large-scale community-based studies have further demonstrated that the gap between subjective and objective sleep duration widens with OSA severity^14,15^.

Objective sleep measures have been shown to be more reliable predictors of all-cause mortality than self-reported sleep duration in individuals with OSA^16^. In the context of hypertension, prior studies have linked short objective sleep duration to an increased risk of hypertension in OSA patients^17,18^. However, these findings are primarily derived from cross-sectional analyses, which limit the ability to establish causal relationships. Prospective studies investigating the impact of objective sleep duration on hypertension incidence in OSA patients remain scarce.

To address this critical gap, our study aims to examine the association between both subjective and objective sleep duration and the prevalence and incidence of hypertension over a five-year follow-up period in individuals with OSA. Using data from the Sleep Heart Health Study—a well-characterized prospective cohort with PSG-derived sleep measurements—we seek to provide novel insights into the role of sleep duration, particularly objective sleep duration, in hypertension risk among OSA patients.

## Methods

### Population

The Sleep Heart Health Study (SHHS) is a multicenter, prospective cohort study designed to investigate the relationship between sleep-disordered breathing and cardiovascular outcomes. The study enrolled 5804 participants aged 40 years and older, with overnight sleep data collected during the initial visit (1995–1998) and reassessed in a subset of 3295 participants during a follow-up visit (2001–2003) using questionnaires and home-based PSG^19–21^.

In this study, we included participants diagnosed with OSA, defined as an apnea-hypopnea index (AHI) of 15 or more events per hour of sleep, characterized by episodes of apnea and hypopnea with ≥ 3% oxygen desaturation or arousal^22^. To examine hypertension incidence, we excluded participants with hypertension at baseline and those with missing hypertension data during the second visit.

### Outcome and main exposure

Hypertension was defined based on blood pressure measurements, medical history, and antihypertensive medication use^23^. During the SHHS home visit, participants underwent a brief health interview, medication assessment, and blood pressure measurement on the night of the sleep exam. Seated blood pressure was measured at home using a conventional mercury sphygmomanometer, with the final value calculated as the average of the second and third readings. Hypertension was defined as systolic blood pressure ≥ 140 mmHg or diastolic blood pressure ≥ 90 mmHg.

The primary exposure, objective sleep duration (PSGTST), was defined as the total sleep time recorded by PSG during a single night at home. Participants were categorized into four groups based on PSGTST: (1) < 5 h, (2) 5–6 h, (3) 6–7 h, and (4) > 7 h, with < 5 h serving as the reference.

The secondary exposure, subjective sleep duration, was assessed using two measures^14^: 1) morning self-reported sleep duration (AMTST): Participants reported their total sleep time upon waking after the PSG night. They were classified into four groups: (1) < 5 h, (2) 5–6 h, (3) 6–7 h, and (4) > 7 h, with < 5 h as the reference. 2) habitual sleep duration: Defined as the weighted average of weekday and weekend sleep durations, calculated as: (weekday habitual sleep duration × 5 + weekend habitual sleep duration × 2) / 7^24^. Participants were grouped as: (1) < 6 h, (2) 6–7 h, (3) 7–8 h, and (4) > 8 h, with < 6 h as the reference.

### Covariates

Baseline assessments collected sociodemographic data and lifestyle habits, including age, gender, ethnicity, body mass index (BMI), smoking status, and daily alcohol and caffeine intake. Medical history included diabetes, cardiovascular and/or cerebrovascular disease (CVD), insomnia, and medication use of lipid-lowering drugs and benzodiazepines within the two weeks prior to baseline. Diabetes was defined by self-reported history or the use of insulin or oral hypoglycemic agents, while CVD history included events such as stroke, congestive heart failure, angina, myocardial infarction, or coronary revascularization. Insomnia was identified through self-reported nighttime symptoms, including difficulty falling asleep, frequent awakenings with trouble returning to sleep, or waking too early and being unable to fall back asleep, occurring 16–30 times per month. Daytime symptoms included persistent fatigue, feeling unrefreshed at least five times per month, or experiencing low energy over the past four weeks^25^.

### Statistical analysis

Continuous variables are presented as means with standard deviation (SD), while categorical variables are reported as counts and percentages. Baseline characteristics were compared across hypertension status using Pearson’s χ² test for categorical variables and the Kruskal-Wallis H test for continuous variables, as none of the continuous variables followed a normal distribution. To assess the association between sleep duration and hypertension prevalence, we employed univariable and multivariable logistic regression models, reporting results as odds ratios (ORs) with 95% confidence intervals (CI). For hypertension incidence, relative risks (RRs) were estimated using a Poisson log-link model with a robust sandwich estimator to account for standard error^26^. Restricted cubic spline (RCS) functions based on the relative regression models were used to assess potential nonlinear associations. Three knots were placed at the 10, 50, and 90th percentiles and robust standard errors were computed for the corresponding models to ensure reliability. All statistical analyses were conducted at a significance level of 0.05 using R (version 4.2.2).

## Results

### Baseline characteristics

**Fig. 1:**
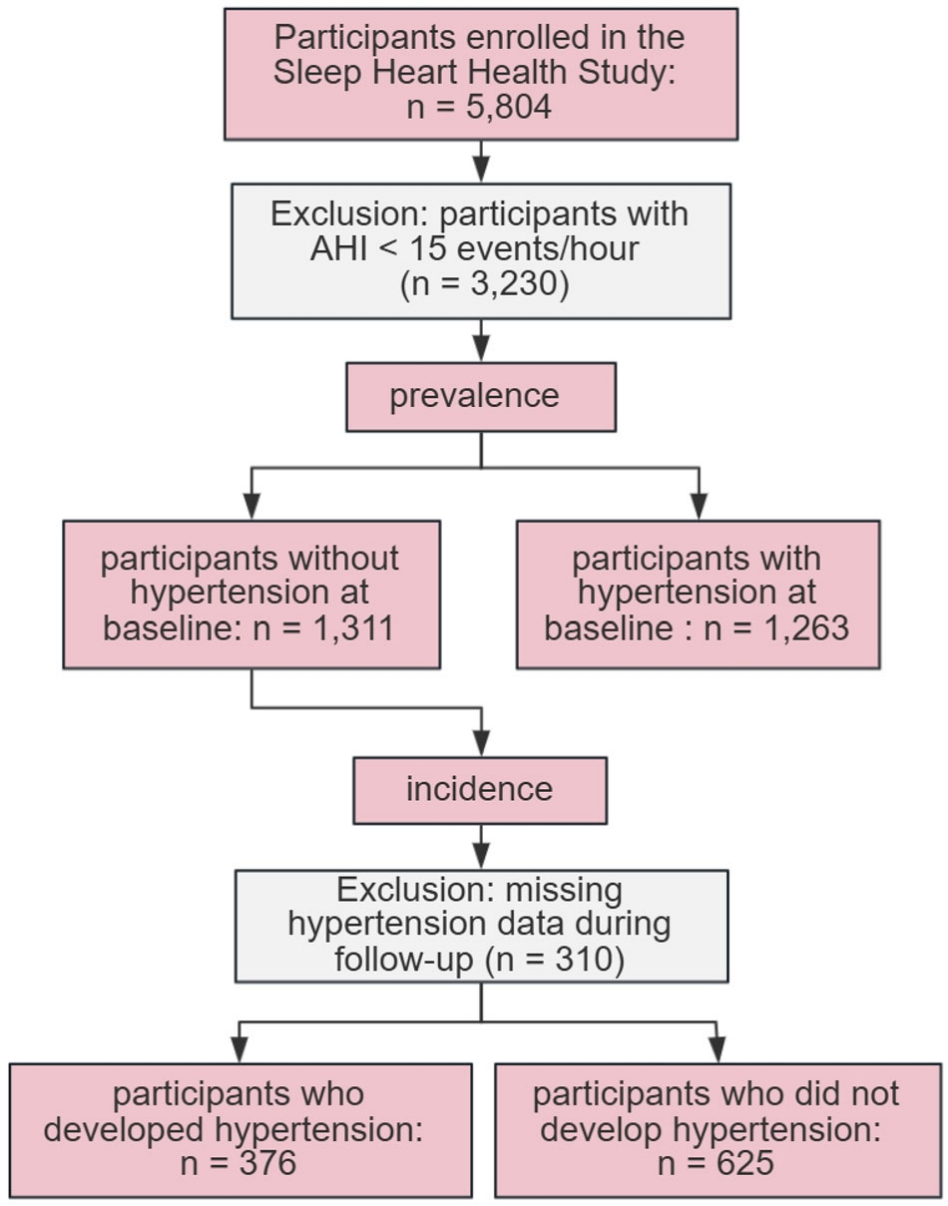
Participant selection process.

After excluding participants with an AHI < 15 events/h (*n* = 3230), a total of 2574 participants were included in the analysis examining the association between sleep duration and hypertension prevalence. For the analysis of hypertension incidence, 1001 participants were included after excluding those with hypertension at baseline (*n* = 1263) and those with missing hypertension data during follow-up (*n* = 310) (Fig. 1).

Table 1 summarized the baseline characteristics according to hypertension status. Objective sleep duration (PSGTST) significantly differed among the three groups (*p* < 0.001): those with baseline hypertension, those who developed hypertension over 5 years, and those who remained free of hypertension. In contrast, the subjective sleep duration measures (AMTST and HABTST) did not show significant differences between the groups.

**Table 1:** Demographics, lifestyle, clinical, and sleep-related features of all patients categorized according to hypertension status.

	Overall	Baseline Hypertension	Incident Hypertension	Normotensive Individuals	*p*
Participants (n)	2574	1263	376	625	
sociodemographics					
Age (years)	65.36 (10.67)	67.74 (10.15)	65.20 (10.01)	62.08 (10.03)	<0.001
BMI (kg/m^2^)	29.56 (5.27)	29.95 (5.58)	29.79 (5.13)	29.00 (4.78)	0.011
Male (%)	1628 (63.2)	776 (61.4)	240 (63.8)	395 (63.2)	0.611
Race					
White	2230 (86.6)	1078 (85.4)	335 (89.1)	571 (91.4)	<0.001
Black	211 (8.2)	141 (11.2)	21 (5.6)	20 (3.2)	
Other	133 (5.2)	44 (3.5)	20 (5.3)	34 (5.4)	
Smoking status					
Never-smoker	1131 (44.2)	575 (45.7)	161 (42.8)	254 (40.8)	0.123
Current smoker	190 (7.4)	76 (6.0)	29 (7.7)	54 (8.7)	
Ex-smoker	1237 (48.4)	607 (48.3)	186 (49.5)	315 (50.6)	
daily alcohol use (%)	1106 (45.9)	502 (42.1)	176 (49.2)	301 (51.6)	<0.001
daily caffeine use (%)	1962 (76.2)	929 (73.6)	297 (79.0)	497 (79.5)	0.006
comorbidity					
diabetes (%)	266 (10.3)	180 (14.3)	31 (8.2)	31 (5.0)	<0.001
CVD history (%)	391 (15.2)	304 (24.1)	34 (9.0)	35 (5.6)	<0.001
lipid-lowering drugs (%)	355 (13.9)	240 (19.0)	36 (9.6)	54 (8.7)	<0.001
insomnia (%)	143 (5.6)	84 (6.8)	15 (4.1)	26 (4.2)	0.028
benzodiazepines use (%)	97 (3.8)	60 (4.8)	16 (4.3)	13 (2.1)	0.019
sleep-related covariates					
PSGTST (h)	5.84 (1.09)	5.72 (1.13)	5.86 (1.05)	6.07 (0.98)	<0.001
AMTST (h)	6.60 (1.47)	6.58 (1.49)	6.57 (1.41)	6.71 (1.40)	0.147
HABTST (h)	7.14 (1.21)	7.10 (1.31)	7.08 (1.10)	7.25 (1.07)	0.074
AHI (events/h)	31.00 (16.10)	32.46 (17.35)	30.16 (16.05)	28.70 (14.07)	<0.001
T90%	6.24 (13.07)	7.66 (15.04)	5.54 (11.22)	4.06 (9.23)	<0.001
Nadir SpO_2_ (%)	82.32 (6.95)	81.81 (7.47)	82.93 (5.90)	83.15 (6.09)	0.001
sleep efficiency (%)	80.87 (11.11)	79.24 (11.73)	81.86 (10.12)	83.40 (9.64)	<0.001
WASO (min)	69.76 (47.19)	75.66 (49.54)	65.73 (45.00)	61.01 (41.74)	<0.001
Arousal index (events/h)	24.47 (12.12)	25.11 (13.15)	24.84 (11.88)	23.14 (10.19)	0.096
% time in REM	18.89 (6.29)	18.59 (6.57)	18.88 (5.92)	19.58 (5.99)	0.011
% time in stages 3 and 4	16.10 (11.60)	16.33 (12.00)	15.24 (11.84)	16.33 (10.89)	0.115

Continuous variables are presented as mean (SD), and categorical variables as n (%).

*PSGTST* total sleep time from polysomnography, *AMTST* morning self-reported total sleep time after polysomnography, *HABTST* self-reported habitual total sleep time, *BMI* body mass index, *AHI* apnea-hypopnea index, *T90%* the percentage of total sleep time during which SpO_2_ was below 90%, *WASO* wake after sleep on, *CVD* cardiovascular and/or cerebrovascular disease, including previous heart failure, coronary heart disease, stroke, *REM* rapid eye movement stage.

### Hypertension prevalence and sleep duration

Table 2 presents the results of logistic regression analyses investigating the relationship between sleep duration and hypertension prevalence.

**Table 2:** Univariable and multivariable logistic regression analysis of the association between objective and subjective sleep duration and the **prevalence** of hypertension (with the category of lowest sleep duration as the reference group).

PSGTST	Participants	No.events	<5 h	5–6 h	6–7 h	>7 h
model 0	2574	1263	1[ref]	0.64 (0.51–0.8)	0.59 (0.48–0.74)	0.51 (0.39–0.68)
model 1	2401	1188	1[ref]	0.71 (0.56–0.9)	0.68 (0.54–0.86)	0.65 (0.48–0.88)
model 2	2355	1164	1[ref]	0.75 (0.58–0.96)	0.69 (0.54–0.88)	0.63 (0.46–0.86)
model 3	2355	1164	1[ref]	0.75 (0.59–0.97)	0.7 (0.55–0.89)	0.63 (0.46–0.87)
AMTST	Participants	No.events	<5 h	5–6 h	6–7 h	>7 h
model 0	2347	1157	1[ref]	0.75 (0.57–0.98)	0.63 (0.49–0.81)	0.78 (0.61–1)
model 1	2189	1087	1[ref]	0.84 (0.63–1.13)	0.76 (0.58–1)	0.88 (0.68–1.15)
model 2	2154	1068	1[ref]	0.91 (0.67–1.24)	0.84 (0.63–1.11)	0.92 (0.7–1.21)
model 3	2154	1068	1[ref]	0.91 (0.67–1.24)	0.83 (0.63–1.11)	0.92 (0.7–1.21)
HABTST	participants	No.events	<6 h	6–7 h	7–8 h	>8 h
model 0	2517	1230	1[ref]	0.7 (0.56–0.88)	0.65 (0.53–0.81)	0.96 (0.73–1.25)
model 1	2347	1156	1[ref]	0.79 (0.62–1)	0.75 (0.6–0.94)	1.02 (0.77–1.36)
model 2	2329	1148	1[ref]	0.88 (0.69–1.14)	0.79 (0.62–1.02)	1.1 (0.81–1.48)
model 3	2329	1148	1[ref]	0.89 (0.69–1.14)	0.79 (0.62–1.02)	1.08 (0.8–1.47)

Associations are presented as odds ratios (95% CI) against the reference group.

model 0: unadjusted.

model 1: adjusted for age, body mass index, gender, race, smoking status, alcohol consumption, and caffeine intake.

model 2: model 1 plus diabetes, cardiovascular and/or cerebrovascular disease, insomnia, and medication use of lipid-lowering drugs and benzodiazepines in the two weeks prior to the baseline assessment.

model 3: model 2 plus apnea-hypopnea index and the percentage of total sleep time during which oxygen saturation was below 90%.

For **PSGTST**, in the unadjusted model, individuals who slept more than five hours had a significantly lower risk of hypertension compared to those who slept less than five hours. Specifically, sleep durations of 5–6 h, 6–7 h, and more than 7 h were associated with a 36% (OR: 0.64, 95% CI: 0.51–0.80), 41% (OR: 0.59, 95% CI: 0.48–0.74), and 49% (OR: 0.51, 95% CI: 0.39–0.68) reduced risk, respectively. These associations remained significant after adjusting for demographic and lifestyle factors (model 1), although the effect size was slightly attenuated: 29% for 5–6 h (OR: 0.71, 95% CI: 0.56–0.90), 32% for 6–7 h (OR: 0.68, 95% CI: 0.54–0.86), and 35% for > 7 h (OR: 0.65, 95% CI: 0.48–0.88). Further adjustment for clinical comorbidities, including diabetes, CVD history, insomnia, and medication use (model 2), resulted in similar reductions: 25% for 5–6 h (OR: 0.75, 95% CI: 0.58–0.96), 31% for 6–7 h (OR: 0.69, 95% CI: 0.54–0.88), and 37% for > 7 h (OR: 0.63, 95% CI: 0.46–0.86). These associations remained consistent in model 3 after additional adjustments for AHI and T90%.

For **AMTST**, model 0 indicated a protective effect for 5–6 h (OR: 0.75, 95% CI: 0.57–0.98) and 6–7 h (OR: 0.63, 95% CI: 0.49–0.81) compared to < 5 h, with a marginally significant association for > 7 h (OR: 0.78, 95% CI: 0.61–1.00). However, these associations weakened after adjusting for demographic, clinical, and OSA-related variables in models 1–3, with no significant associations remaining in the fully adjusted model.

For **HABTST**, in model 0, sleep durations of 6–7 h (OR: 0.70, 95% CI: 0.56–0.88) and 7–8 h (OR: 0.65, 95% CI: 0.53–0.81) were associated with a lower risk of hypertension compared to < 6 h. However, longer sleep durations (>8 h) did not show a significant protective effect. These associations weakened after further adjustments and became nonsignificant in the fully adjusted models.

To address potential bias from reference group selection, we reanalyzed the data using the longest sleep duration category as the reference. In this analysis, <5 h of PSGTST was consistently associated with a higher prevalence of hypertension across all models, with ORs ranging from 1.54–1.95. In contrast, the association between AMTST and hypertension weakened and lost statistical significance after adjustment. For HABTST, sleeping 7–8 h demonstrated a protective effect against hypertension compared to > 8 h in all models. However, sleep duration of < 6 h showed no significant difference in hypertension prevalence relative to the reference group, suggesting a nonlinear relationship between HABTST and hypertension prevalence (Supplementary Table 1).

**Fig. 2:**
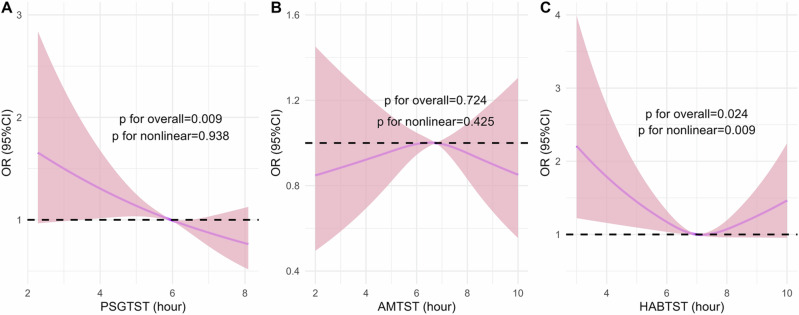
Restricted cubic spline analysis of the association between different sleep duration categories and the prevalence of hypertension. This figure illustrates the restricted cubic spline analysis assessing the relationship between sleep duration and the prevalence of hypertension, after adjusting for demographic, lifestyle, and clinical factors. **A** shows objectively measured sleep duration (PSGTST), while **B** and **C** show subjectively measured sleep duration (AMTST and HABTST, respectively). The solid purple line represents the estimated odds ratio (OR), and the shaded pink area indicates the 95% confidence interval (CI). The horizontal dashed line represents an OR of 1.

The **RCS analysis** revealed a significant negative correlation between PSGTST and hypertension prevalence (*p* = 0.009), with no evidence of nonlinearity (*p* = 0.938). In contrast, AMTST showed no significant association with hypertension prevalence (p for overall = 0.724, p for nonlinearity = 0.425). For HABTST, a U-shaped relationship was observed (p for overall = 0.024, p for nonlinearity = 0.009), suggesting that both very short and long habitual sleep durations may be linked to higher hypertension prevalence (Fig. 2). A sensitivity analysis excluding six participants using continuous positive airway pressure (CPAP) or a mouthpiece for sleep apnea treatment did not materially alter the association between sleep duration and hypertension prevalence (Supplementary Fig. 1).

### Hypertension incidence and sleep duration

After a follow-up period of 5.25 ± 0.27 years, 376 out of 1001 OSA patients developed hypertension. Table 3 summarizes the relative risks (RR) associated with different sleep duration categories and hypertension incidence.

**Table 3:** Univariable and multivariable Poisson regression analysis of the association between objective and subjective sleep duration and the **incidence** of hypertension (with the category of lowest sleep duration as the reference group).

PSGTST	Participants	No.events	<5 h	5–6 h	6–7 h	>7 h
model 0	1001	376	1[ref]	0.77 (0.58–1.02)	0.67 (0.51–0.89)	0.66 (0.46–0.94)
model 1	939	358	1[ref]	0.81 (0.6–1.09)	0.72 (0.54–0.97)	0.72 (0.49–1.05)
model 2	924	351	1[ref]	0.81 (0.6–1.09)	0.72 (0.54–0.98)	0.72 (0.48–1.05)
model 3	924	351	1[ref]	0.81 (0.6–1.09)	0.73 (0.54–0.98)	0.72 (0.49–1.05)
AMTST	Participants	No.events	<5 h	5–6 h	6–7 h	>7 h
model 0	913	342	1[ref]	0.79 (0.55–1.13)	0.84 (0.61–1.17)	0.78 (0.57–1.08)
model 1	856	325	1[ref]	0.76 (0.53–1.11)	0.85 (0.61–1.19)	0.76 (0.55–1.07)
model 2	845	321	1[ref]	0.78 (0.53–1.14)	0.86 (0.61–1.21)	0.76 (0.55–1.07)
model 3	845	321	1[ref]	0.78 (0.53–1.14)	0.86 (0.61–1.21)	0.76 (0.54–1.07)
HABTST	Participants	No.events	<6 h	6–7 h	7–8 h	>8 h
model 0	988	371	1[ref]	0.72 (0.54–0.96)	0.75 (0.58–0.98)	0.67 (0.46–0.97)
model 1	926	353	1[ref]	0.76 (0.57–1.01)	0.82 (0.63–1.08)	0.73 (0.5–1.06)
model 2	918	349	1[ref]	0.76 (0.56–1.02)	0.82 (0.62–1.09)	0.73 (0.49–1.06)
model 3	918	349	1[ref]	0.76 (0.56–1.02)	0.82 (0.62–1.09)	0.73 (0.49–1.06)

Associations are presented as relative risks (95% CI) against the reference group.

model 0: unadjusted.

model 1: adjusted for age, body mass index, gender, race, smoking status, alcohol consumption, and caffeine intake.

model 2: model 1 plus diabetes, cardiovascular and/or cerebrovascular disease, insomnia, and medication use of lipid-lowering drugs and benzodiazepines in the two weeks prior to the baseline assessment.

model 3: model 2 plus apnea-hypopnea index and the percentage of total sleep time during which oxygen saturation was below 90%.

For **PSGTST**, In the unadjusted model, individuals who slept more than five hours had a significantly lower risk of developing hypertension compared to those sleeping less than five hours. Specifically, sleeping 5–6 h was associated with a 23% lower risk (RR: 0.77, 95% CI: 0.58–1.02), 6–7 h with a 33% reduction (RR: 0.67, 95% CI: 0.51–0.89), and more than 7 h with a 34% reduction (RR: 0.66, 95% CI: 0.46–0.94). After adjusting for demographic and lifestyle factors in model 1, the reductions were 19% for 5–6 h (RR: 0.81, 95% CI: 0.6–1.09), 28% for 6–7 h (RR: 0.72, 95% CI: 0.54–0.97), and 28% for > 7 h (RR: 0.72, 95% CI: 0.49–1.05). These risk reductions remained stable across model 2 and 3.

For **AMTST**, in the unadjusted model, no significant associations were observed across sleep duration categories. Compared to < 5 h, the RRs were 0.79 (95% CI: 0.55–1.13) for 5–6 h, 0.84 (95% CI: 0.61–1.17) for 6–7 h, and 0.78 (95% CI: 0.57–1.08) for > 7 h. These associations remained largely unchanged after adjusting for demographic, clinical, and sleep-related factors in models 1–3.

For **HABTST**, model 0 showed that sleep durations of 6–7 h (RR = 0.72, 95% CI: 0.54–0.96), 7–8 h (RR = 0.75, 95% CI: 0.58–0.98), and > 8 h (RR = 0.67, 95% CI: 0.46–0.97) were significantly associated with a lower risk of hypertension compared to < 6 h. However, after adjusting for potential confounders in models 1–3, the associations were attenuated.

When using the longest sleep duration category as the reference, individuals with the shortest PSGTST (<5 h) consistently exhibited the highest risk of developing hypertension across all models, despite a reduction in statistical significance after full adjustment (model 3: RR = 1.39, 95% CI: 0.95–2.06). Similar patterns were observed for AMTST and HABTST, where the shortest sleep duration group was associated with an increased risk of developing hypertension (Supplementary Table 2).

**Fig. 3:**
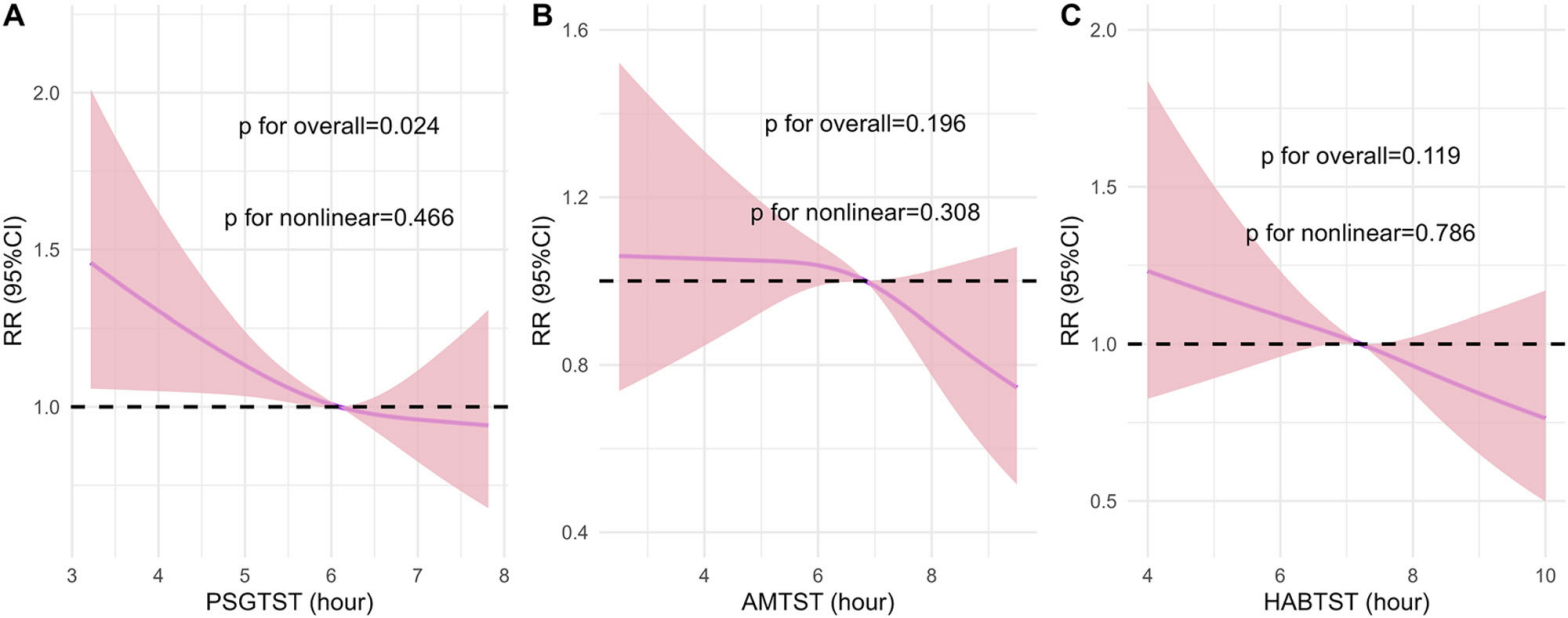
Restricted cubic spline analysis of the association between different sleep duration categories and the incidence of hypertension. This figure illustrates the restricted cubic spline analysis assessing the relationship between sleep duration and the incidence of hypertension, after adjusting for demographic, lifestyle, and clinical factors. **A** shows objectively measured sleep duration (PSGTST), while **B** and **C** show subjectively measured sleep duration (AMTST and HABTST, respectively). The solid purple line represents the estimated relative risks (RR), and the shaded pink area indicates the 95% confidence interval (CI). The horizontal dashed line represents an RR of 1.

The **RCS analysis** revealed a significant negative linear correlation between PSGTST and hypertension incidence (p for overall = 0.024), with no evidence of nonlinearity (*p* = 0.466). AMTST and HABTST showed weaker associations with hypertension incidence (*p* = 0.196 and *p* = 0.119 for overall, and *p* = 0.308 and *p* = 0.786 for nonlinearity, respectively) (Fig. 3). A sensitivity analysis excluding 39 participants who received sleep apnea treatment at baseline and during follow-up did not alter the overall association trends (Supplementary Fig. 2).

## Discussion

This study investigated the association between objective and subjective sleep duration and the prevalence and incidence of hypertension in individuals with OSA. Our findings indicate that objective sleep duration (PSGTST) shows a clearer and more consistent linear negative correlation with hypertension prevalence and incidence compared to subjective sleep duration measures (AMTST and HABTST).

In individuals with OSA, a discrepancy often exists between subjective and objective sleep duration, and this gap tends to widen with increasing OSA severity^14,15^. Patients with more severe OSA typically experience reduced objective sleep duration^27^. Additionally, a subset of OSA patients who also suffers from insomnia may underestimate their sleep duration^10–13^. Given these variations, relying solely on self-reported sleep duration could introduce recall bias and obscure true associations between sleep and cardiovascular outcomes. Therefore, assessing both objective and subjective sleep measures is essential for accurately evaluating the impact of sleep duration on hypertension risk in patients with OSA.

Previous research on the relationship between sleep duration and hypertension in OSA patients remains limited. A small study previously found that a combination of severe OSA and short objective sleep duration was associated with increased hypertension prevalence^17^. A subsequent large cross-sectional study confirmed that OSA patients with short objective sleep duration, rather than short subjective sleep duration, had an increased risk of hypertension^18^. Later, Drager et al. argued that OSA, rather than short objective sleep duration, is a risk factor for hypertension^28^. The cross-sectional nature of these studies likely contributed to the variability in findings. Currently, no prospective studies have have examined how sleep duration impacts hypertension incidence in OSA patients over time. Our study fills this gap by investigating the association between different sleep duration measures and both hypertension prevalence and incidence over a five-year follow-up period.

Our results demonstrate that PSGTST showed a significant and consistent inverse linear relationship with both hypertension prevalence and incidence, suggesting that longer objective sleep duration may reduce hypertension risk in OSA patients. In contrast, HABTST displayed a U-shaped association with hypertension prevalence, where both short and long sleep durations were associated with elevated risk. For hypertension incidence, HABTST showed a weaker but still negative trend. AMTST demonstrated the weakest and least significant associations with both outcomes.

The association between sleep duration and hypertension incidence has been reported in multiple studies and recently summarized in systematical reviews and meta-analyses^8,9^. Overall, the majority of research indicates that shorter sleep duration is associated with an increased risk of both hypertension prevalence and incidence^29^. However, the effect of longer sleep duration on hypertension risk remains inconsistent. Notably, most prior studies have relied on subjective sleep duration measures, which may explain the heterogeneity in findings. In contrast, objective sleep metrics have recently gained attention as more reliable indicators of cardiometabolic health. For instance, increased objective sleep duration has been associated with reduced all-cause and CVD mortality, whereas self-reported usual sleep duration follows a J-shaped relationship with these outcomes^30^. In patients with insomnia, objective sleep duration is more strongly linked to an increased risk of incident hypertension^31^ and CVD incidence^32^ compared to subjective sleep duration. Similarly, in the OSA population, short objective sleep duration, rather than subjective sleep duration, has been significantly linked to increased all-cause mortality^16^ and hypertension prevalence^18^.

Our study builds upon prior research by emphasizing that, in patients with OSA, objective sleep duration demonstrates a more significant and linear relationship with both hypertension prevalence and incidence. This may be due to the fact that OSA patients tend to misperceive their sleep duration, which could attenuate the strength of associations based on subjective reports. Nevertheless, our findings do not contradict the well-established link between subjective short sleep duration and hypertension risk. For example, HABTST— defined as the average weekly self-reported sleep duration— displayed a U-shaped relationship with hypertension prevalence, with increased risk at durations shorter than 7 h. Similarly, individuals reporting less than 6 h of HABTST had the highest risk of developing hypertension. However, the overall associations between subjective sleep duration and hypertension were weaker and less consistent than those observed with PSGTST. Taken together, these results suggest that while subjective short sleep duration is a relevant risk factor, objective sleep duration may serve as a more robust and reliable indicator of hypertension risk among individuals with OSA.

This study has several strengths. By incorporating both objective and subjective sleep measures, we provide a more holistic evaluation of sleep duration’s impact on hypertension risk in OSA patients. Moreover, the prospective design of the study enabled us to utilize relative risks, rather than odds ratios, to more accurately explore the temporal relationship between sleep duration and the development of hypertension over time. Additionally, we adjusted for key confounders, including insomnia, cardiovascular disease, diabetes, and other hypertension risk factors, which strengthens the validity of our findings.

However, there are certain limitations to consider. Objective sleep duration was based on a single-night PSG, which may not fully capture night-to-night variability, potentially leading to misclassification of OSA severity and sleep duration^33^. While habitual total sleep time offers valuable insights into long-term sleep patterns, it is prone to recall bias. To enhance the accuracy of sleep duration assessments and better understand its relationship with hypertension risk, future studies should incorporate both actigraphy and sleep diaries to allow for repeated measurements and better capture individual sleep patterns over time. Additionally, this study focused solely on total sleep time, but other sleep parameters—such as sleep efficiency, slow-wave sleep percentage, and sleep timing—may also influence cardiovascular outcomes^34–39^. Future research should explore how these aspects of sleep architecture interact with OSA severity to better understand their impact on cardiovascular health in OSA patients.

In conclusion, our study provides strong evidence that objective sleep duration has a clearer and more consistent linear negative correlation with both hypertension prevalence and incidence compared to subjective sleep duration measures in OSA patients. These findings highlight the importance of incorporating objective sleep metrics into clinical assessments and suggest that improving objective sleep duration may be an effective strategy for reducing hypertension risk in this population.

## Supplementary information

Supplementary material

## Data Availability

The data are available via the National Sleep Research Resource (https://sleepdata.org/).
