# Supplementary material for: Association between sleep duration and hypertension risk in patients with obstructive sleep apnea
Source: NPJ Prim Care Respir Med. 2025 Apr 28;35:26. doi: 10.1038/s41533-025-00429-7 (PMC12037815; doi:10.1038/s41533-025-00429-7)
Supplement: Supplementary file 1 — Supplementary material [file 41533_2025_429_MOESM1_ESM.doc]

**supplementary materials**

**Supplementary Table 1.** Univariable and multivariable logistic regression analysis of the association between objective and subjective sleep duration and the **prevalence** of hypertension (with the highest sleep duration category as the reference group).

| PSGTST | Participants | No.events | >7 hours | 6-7 hours | 5-6 hours | <5 hours |
| --- | --- | --- | --- | --- | --- | --- |
| model 0 | 2574 | 1263 | 1[ref] | 1.16 (0.9-1.49) | 1.26 (0.97-1.62) | 1.95 (1.48-2.59) |
| model 1 | 2401 | 1188 | 1[ref] | 1.05 (0.8-1.39) | 1.1 (0.83-1.45) | 1.54 (1.14-2.09) |
| model 2 | 2355 | 1164 | 1[ref] | 1.1 (0.83-1.47) | 1.19 (0.89-1.6) | 1.59 (1.16-2.19) |
| model 3 | 2355 | 1164 | 1[ref] | 1.11 (0.83-1.47) | 1.19 (0.89-1.6) | 1.58 (1.15-2.17) |
| AMTST | Participants | No.events | >7 hours | 6-7 hours | 5-6 hours | <5 hours |
| model 0 | 2347 | 1157 | 1[ref] | 0.8 (0.66-0.98) | 0.96 (0.76-1.2) | 1.28 (1-1.63) |
| model 1 | 2189 | 1087 | 1[ref] | 0.86 (0.69-1.07) | 0.95 (0.75-1.22) | 1.13 (0.87-1.47) |
| model 2 | 2154 | 1068 | 1[ref] | 0.91 (0.73-1.14) | 0.99 (0.76-1.28) | 1.09 (0.82-1.43) |
| model 3 | 2154 | 1068 | 1[ref] | 0.91 (0.72-1.14) | 0.99 (0.77-1.28) | 1.09 (0.83-1.44) |
| HABTST | Participants | No.events | >8 hours | 7-8 hours | 6-7 hours | <6 hours |
| model 0 | 2517 | 1230 | 1[ref] | 0.68 (0.53-0.87) | 0.73 (0.57-0.95) | 1.04 (0.8-1.36) |
| model 1 | 2347 | 1156 | 1[ref] | 0.74 (0.56-0.96) | 0.77 (0.59-1.02) | 0.98 (0.74-1.31) |
| model 2 | 2329 | 1148 | 1[ref] | 0.72 (0.55-0.95) | 0.81 (0.61-1.07) | 0.91 (0.67-1.23) |
| model 3 | 2329 | 1148 | 1[ref] | 0.73 (0.56-0.97) | 0.82 (0.62-1.09) | 0.92 (0.68-1.25) |
| Associations are presented as odds ratios (95% CI) against the reference group.  model 0: unadjusted;  model 1: adjusted for age, body mass index, gender, race, smoking status, alcohol consumption, and caffeine intake;  model 2: model 1 plus diabetes, cardiovascular and/or cerebrovascular disease, insomnia, and medication use of lipid-lowering drugs and benzodiazepines in the two weeks prior to the baseline assessment;  Model 3: model 2 plus apnea-hypopnea index and the percentage of total sleep time during which oxygen saturation was below 90%. | | | | | | |

**supplementary Table 2.** Univariable and multivariable logistic regression analysis of the association between objective and subjective sleep duration and the **incidence** of hypertension (with the highest sleep duration category as the reference group).

| PSGTST | Participants | No. events | >7 hours | 6-7 hours | 5-6 hours | <5 hours |
| --- | --- | --- | --- | --- | --- | --- |
| model 0 | 1001 | 376 | 1[ref] | 1.01 (0.74-1.42) | 1.16 (0.84-1.63) | 1.51 (1.06-2.17) |
| model 1 | 939 | 358 | 1[ref] | 1 (0.71-1.42) | 1.12 (0.8-1.6) | 1.39 (0.95-2.04) |
| model 2 | 924 | 351 | 1[ref] | 1.01 (0.72-1.45) | 1.13 (0.8-1.62) | 1.4 (0.96-2.06) |
| model 3 | 924 | 351 | 1[ref] | 1.01 (0.72-1.45) | 1.13 (0.8-1.62) | 1.39 (0.95-2.06) |
| AMTST | Participants | No. events | >7 hours | 6-7 hours | 5-6 hours | <5 hours |
| model 0 | 913 | 342 | 1[ref] | 1.08 (0.83-1.4) | 1.01 (0.74-1.37) | 1.29 (0.93-1.77) |
| model 1 | 856 | 325 | 1[ref] | 1.11 (0.85-1.46) | 1 (0.72-1.37) | 1.31 (0.94-1.81) |
| model 2 | 845 | 321 | 1[ref] | 1.13 (0.86-1.48) | 1.02 (0.74-1.4) | 1.32 (0.93-1.83) |
| model 3 | 845 | 321 | 1[ref] | 1.13 (0.86-1.48) | 1.02 (0.74-1.4) | 1.32 (0.93-1.84) |
| HABTST | Participants | No. events | >8 hours | 7-8 hours | 6-7 hours | <6 hours |
| model 0 | 988 | 371 | 1[ref] | 1.12 (0.79-1.62) | 1.07 (0.75-1.57) | 1.49 (1.03-2.18) |
| model 1 | 926 | 353 | 1[ref] | 1.12 (0.79-1.63) | 1.04 (0.72-1.52) | 1.37 (0.94-2.02) |
| model 2 | 918 | 349 | 1[ref] | 1.13 (0.79-1.64) | 1.04 (0.72-1.53) | 1.37 (0.94-2.04) |
| model 3 | 918 | 349 | 1[ref] | 1.13 (0.79-1.64) | 1.05 (0.72-1.54) | 1.38 (0.94-2.05) |
| Associations are presented as relative risks (95% CI) against the reference group.  model 0: unadjusted;  model 1: adjusted for age, body mass index, gender, race, smoking status, alcohol consumption, and caffeine intake;  model 2: model 1 plus diabetes, cardiovascular and/or cerebrovascular disease, insomnia, and medication use of lipid-lowering drugs and benzodiazepines in the two weeks prior to the baseline assessment;  model 3: model 2 plus apnea-hypopnea index and the percentage of total sleep time during which oxygen saturation was below 90%. | | | | | | |

Supplementary Figure 1. Restricted cubic spline analysis of the association between different sleep duration categories and the **prevalence** of hypertension (**excluding individuals receiving treatment for OSA**).


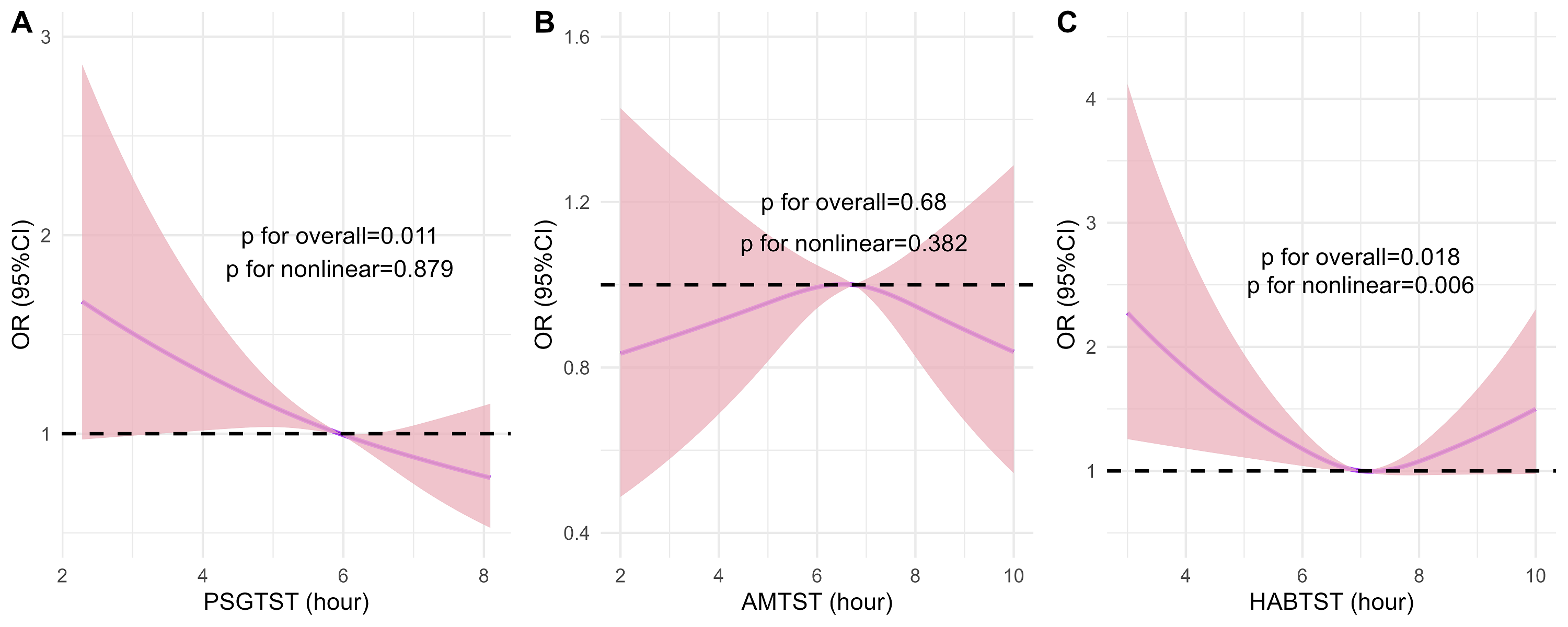


This figure illustrates the restricted cubic spline analysis assessing the relationship between sleep duration—measured objectively (PSGTST) and subjectively (AMTST, HABTST)—and the prevalence of hypertension, after adjusting for demographic, lifestyle, and clinical factors. The solid purple line represents the estimated odds ratio (OR), while the shaded pink area shows the 95% confidence interval (CI) surrounding these estimates. The horizontal dashed line represents an OR of 1.

Supplementary Figure 2. Restricted cubic spline analysis of the association between different sleep duration categories and the **incidence** of hypertension (**excluding individuals receiving treatment for OSA**).


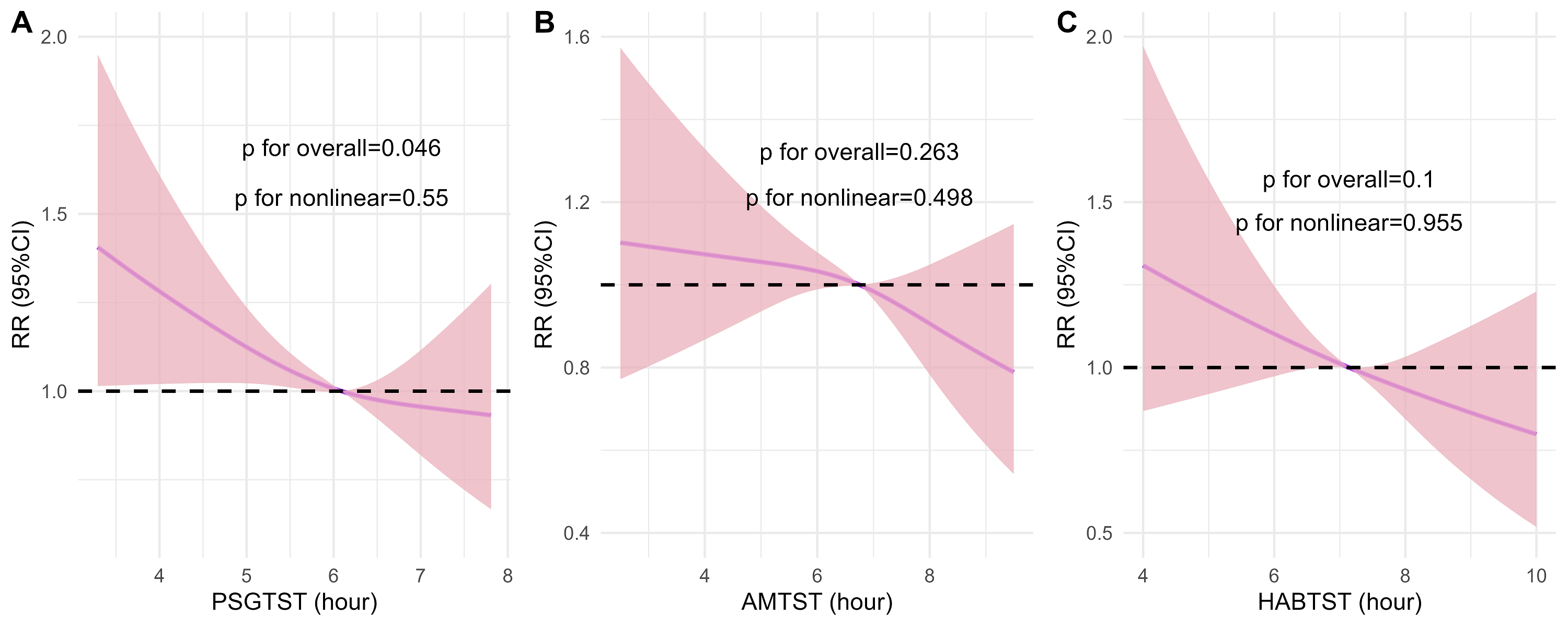


This figure illustrates the restricted cubic spline analysis assessing the relationship between sleep duration—measured objectively (PSGTST) and subjectively (AMTST, HABTST)—and the incidence of hypertension, after adjusting for demographic, lifestyle, and clinical factors. The solid purple line represents the estimated relative risks (RR), while the shaded pink area shows the 95% confidence interval (CI) surrounding these estimates. The horizontal dashed line represents an RR of 1.
